# A Chemiluminescence Enzyme Immunoassay Based on Biotinylated Nanobody and Streptavidin Amplification for Diazinon Sensitive Quantification

**DOI:** 10.3390/bios13060577

**Published:** 2023-05-25

**Authors:** Pengyan Guo, Kaiyin Huang, Zijian Chen, Zhenlin Xu, Aifen Ou, Qingchun Yin, Hong Wang, Xing Shen, Kai Zhou

**Affiliations:** 1 Guangdong Provincial Key Laboratory of Food Quality and Safety, South China Agricultural University, Guangzhou 510642, China; 2 Institute of Jiangxi Oil-Tea Camellia, Jiujiang University, Jiujiang 332000, China; 3 School of Food Science and Health Preserving, Guangzhou City Polytechnic, Guangzhou 510006, China; 4 Key Laboratory of Tropical Fruits and Vegetables Quality and Safety for State Market Regulation, Hainan Institute for Food Control, Haikou 570314, China

**Keywords:** diazinon, nanobody, phage display, indirect competitive chemiluminescence enzyme immunoassay, signal amplification

## Abstract

The advantages of genetic modification and preferable physicochemical qualities make nanobody (Nb) easy to develop a sensitive and stable immunosensor platform. Herein, an indirect competitive chemiluminescence enzyme immunoassay (ic-CLEIA) based on biotinylated Nb was established for the quantification of diazinon (DAZ). The anti-DAZ Nb, named Nb-EQ1, with good sensitivity and specificity, was obtained from an immunized library via a phage display technique, where the molecular docking results indicated that the hydrogen bond and hydrophobic interactions between DAZ and complementarity-determining region 3 and framework region 2 in Nb-EQ1 played a critical role in the Nb-DAZ affinity processes. Subsequently, the Nb-EQ1 was further biotinylated to generate a bi-functional Nb-biotin, and then an ic-CLEIA was developed for DAZ determination via signal amplification of the biotin–streptavidin platform. The results showed that the proposed method based on Nb-biotin had a high specificity and sensitivity to DAZ, with a relative broader linear range of 0.12–25.96 ng/mL. After being 2-folds dilution of the vegetable samples matrix, the average recoveries were 85.7–113.9% with a coefficient of variation of 4.2–19.2%. Moreover, the results for the analysis of real samples by the developed ic-CLEIA correlated well with that obtained by reference method GC-MS (R^2^ ≥ 0.97). In summary, the ic-CLEIA based on biotinylated Nb-EQ1 and streptavidin recognition demonstrated itself to be a convenient tool for the quantification of DAZ in vegetables.

## 1. Introduction

Immunoassay has been widely used in the rapid detection of pesticides in agricultural products due to its low cost, simple operation, high sensitivity, and good specificity [1,2]. The key criterion of immunoassays for the detection of small chemicals is the use of highly selective antibodies without obvious cross-reactivity to other similar chemicals [3]. However, polyclonal and monoclonal antibodies (pAbs and mAbs), the widely used two types of antibodies, are raised by a traditional strategy of preparing immunogens and coating antigens, which are born with defects. The instability of pAb production results in an obvious difference between batches, cannot be replicated on a large scale, and is prone to the production of non-specific antibodies [4]. mAbs are generated by hybridoma cells with high specificity and yields that are increasingly being used in immunoassays, but they demand complex preparation procedures and a strict preservation condition [5]. Moreover, the production of mAb against the small molecular weight of the analytes is hard due to the extremely low rate of the immune response in experimental animals [6]. In practice, in addition to the consideration of the novel design and synthesis of haptens for the generation of antibodies [7], plenty of mice need to be immunized to improve the success rate of the immune response due to the individual difference.

Recently, a kind of single domain antibody, named nanobody (Nb), possesses a genetic modification advantage and preferable physicochemical qualities that make it easy to develop a sensitive and stable immunosensor platform for the determination of contaminants in food and the environment [8,9]. It is usually obtained by cloning the variable domain of the heavy chain antibodies (VHH) of camelids or sharks, owing to their preferable properties, such as small size, elevated solubility, and admirable thermal stability in comparison with conventional antibodies [10]. Moreover, the advantages of genetic modification and expression and a good chemical stability make the Nb easy to form various bifunctional Nb-based fusion proteins and Nb-based binding proteins that can build flexible immunosensor [11,12]. Up to now, nanobody-based biosensors, as promising technical intercessions, have been widely applied for environmental monitoring and food safety redetermination, for example, the produced nanobody exhibited a good sensitivity against anti-2,4-dichlorophenoxyacetic acid, which had a better specificity than the rabbit pAb [13]; in the determination of aflatoxin genetic fungi, the sensitivity of the immobilization Nb mode was at least 500 times higher than that of the immobilization IgG mode [14].

In addition to the high quality of antibodies, another key criterion of immunoassays is a novel and highly sensitive immunochemical design for the detection of small-molecule analytes [15]. Immunoassay that combined with chemiluminescence technology is increasingly considered a viable alternative to an enzyme-linked immunoassay (ELISA) owing to its broad dynamic range, high signal intensity, and better specificity [16]. The detection principle is based on changes in the luminescence signal generated by the specific recognition between antigen and antibody. Various types of chemiluminescence immunoassay, such as chemiluminescence enzyme immunoassay (CLEIA) [17], direct chemiluminescence immunoassay (DCLIA) [18], and electrochemiluminescence immunoassay (ECLIA) [19], have been developed for the determination of various analytes.

Diazinon (DAZ) is a widely used organophosphate insecticide in both agricultural and non-agricultural applications that results in it being frequently detected in the environment and in agricultural products, such as fruits and vegetables. It acts as a cholinesterase inhibitor, and it kills pests via poisoning their central nervous system [20]. Currently, many studies have demonstrated DAZ’s immunotoxicity, cytotoxicity, and genotoxicity to animals [21]. Due to the potential toxicity of DAZ to human health, DAZ residues in food products and environments have been strictly monitored and evaluated, and the strict maximum residue limits (MRLs) in different areas have been set up, ranging from 0.01 mg/kg to 0.05 mg/kg for fruit and vegetables [22].

In our previous study [20], a novel heterology coating strategy was successfully used for screening mAb against DAZ, and the developed indirect competitive enzyme-linked immunoassay (ic-ELISA) exhibited a good limit of detection (LOD) of 8 pg/mL for DAZ determination, but the narrow linear range of 0.16 ng/mL to 2.10 ng/mL was inconvenient for the demand of the quantification of DAZ residues in actual samples. Herein, we produced the Nb-specific recognition of DAZ using the phage display technique, and then an ic-CLEIA platform biotinylated Nb-EQ1 and streptavidin recognition was constructed for DAZ qualification with an acceptable satisfactory linear response range.

## 2. Materials and Methods

### 2.1. Materials

The anti-DAZ mAb, coating antigen DAZ-A-BSA, and immunogen DAZ-B-LF were prepared by our laboratory [20]. The helper phage, vector, and *E. coli* related to this study were the same as those previously reported by our laboratory [23]. Standard regents of DAZ and its structural analogues were supplied by Macklin Chemical Technology Co., Ltd. (Shanghai, China). Protein A/G resins and rabbit anti-VHH-HRP were purchased from TransGen Biotech Co., Ltd. (Beijing, China). The Total RNA kit, cDNA synthesis kit, gel extraction kit, and PCR purification kit were purchased from TNAGEN Co., Ltd. (Beijing, China). Sulfo-NHS biotin, streptavidin labeled horseradish peroxidase (SA-HRP), enhanced chemiluminescent (ECL), and penicillin streptomycin were purchased from Thermo Fisher (Shanghai, China). Complete and incomplete Freund’s adjuvants were obtained from Sigma (St. Louis, MO, USA). The other reagents purchased from Sigma-Aldrich Co., Ltd. (Shanghai, China) were of analytical reagent. The 96-well polystyrene microtiter plates were obtained from Yijiamei Industrial Co., Ltd. (Xiamen, China).

### 2.2. Preparation of Anti-DAZ Nb

The anti-DAZ Nb was prepared as previously described [23]. Briefly, a three-year-old male Bactrian camel was immunized with DAZ-B-LF biweekly, and the immune serum was collected for Nbs library construction. Fresh blood was collected and used for lymphocyte isolation; the processes of RNA extraction and cDNA synthesis were conducted by referring to the directions on the kits, respectively. Then, the VHH genes were amplified by two-step nested PCR; the primers are shown in Table S1. The expression vector PComb3XSS-VHH was constructed after digestion with the *SfiI* enzyme separately and was then transformed into *E. coli* TG1 competent cells via electro transformation. Ten clones were selected randomly and sequenced to evaluate the insertion rate of the library and library diversity. Phage display technology was adopted to screen the positive clone from the constructed nanobody library above, and the selected specific phage clones were sent for DNA sequencing [24]. The specific pComb3xss-VHH plasmid was transferred into *E. coli* BL21(DE)3 cells via electrotransformation (GenePulser XcellTM, BIO-RAD, Hercules, CA, USA) at 2.5 kV, and the prepared cells were gradient dilution and then cultured on LB-ampicillin agar plates overnight at 37 °C. After gene sequencing, the positive clone named Nb-EQ1 was induced to express anti-DAZ Nb by LB culture containing isopropyl β-D-1-thiogalactopyranoside (1 mM). The anti-DAZ Nb was extracted and purified by Ni-NTA affinity chromatography, then identified by SDS-PAGE. The target protein content was detected using a NanoDrop 2000C system (Thermo Scientific, Waltham, MA, USA) after concentration.

### 2.3. Characteristics of Nb-EQ1

The binding activity of prepared Nb-EQ1 against DAZ was detected by ic-ELISA according to the previously study [20]. The difference is that HRP-conjugated goat anti-mouse IgG was replaced by HRP-conjugated rabbit anti-VHH.

The thermostability, organic solvent, and acid tolerance of Nb-EQ1 were tested and compared with DAZ-mAb. Briefly, the Nb-EQ1 and DAZ-mAb were (i) heated in water baths with a temperature of 20 °C, 35 °C, 50 °C, 65 °C, 80 °C, and 95 °C for 5 min, (ii) diluted to the working concentration with different contents (0%, 10%, 20%, 40%, and 50%) of methanol, acetonitrile, and acetone, and (iii) diluted to the working concentration with PBS solutions with different pH values (1.4, 2.4, 3.4, 4.4, 5.4, 6.4, 7.4, 8.4, 9.4, and 10.4). The binding activities of antibodies against DAZ were determined to estimate the endurance capacity, and the binding activities of untreated Nb-EQ1 against DAZ were regarded as 100% in testing the thermostability and organic solvent resistance.

Swiss Model software was used for homology modeling, and the template with a high homology and coverage was selected as the model. The rationality of the 3D nanobody model obtained by the PROCHECK online database was evaluated. The molecule docking between DAZ and the CDR region of Nb-EQ1 was analyzed using Lead IT 2.3.2-Linux-x64 software on the Enthalpy and Entropy way (Hybrid Approach).

### 2.4. Development of Nb-EQ1 Based ic-CLEIA

Preparation of biotinylated Nb-EQ1: 800 μL purified Nb-EQ1 (1 mg/mL) was added into 1 mg/mL sulfo-NHS biotin dropwise. The mixture was stirred at 4 °C for 12 h, followed the dialysis (molecular weight cutoff: 3 kD) for a total of 3 days in PBS buffers (0.01 M). Finally, the biotinylated Nb-EQ1 was stored at 4 °C until used.

The procedures of the ic-CLEIA were similar to that of the ic-ELISA. The 96-well polystyrene microplates (Yijiamei Industrial Co., Ltd, Xiamen, China) were coated with DAZ-A-BSA at 4 °C overnight, and the blocking agent was 2% BSA. The optimized concentration of biotinylated Nb-EQ1 (50 μL/well) and serial concentrations of DAZ (50 μL/well) were added sequentially and incubated at 37 °C for 40 min. After competitive reaction and five washes with PBST, 100 μL of diluted SA-HRP was added to each well and then incubated for 40 min at 37 °C. After washing 5 times, 100 μL of the ECL illuminant was added to each well. In order to save time when filling the microtiter plate, the 8-channel pipette was used for adding ECL illuminant, and this immediately measured one row of 96-well plates each time in practice. The chemiluminescence intensity was determined using a SpectraMax i3 (Molecular Devices, Shanghai, China) immediately.

To achieve the best performance, the optimal anti-DAZ Nb and DAZ-A-BSA concentrations were tested using the Checkerboard titration method [25], and the other parameters, including PBS buffer (from 5 mM to 80 mM), pH (from 5.4 to 9.4), and Twen-80 (from 0 to 0.5‰), were studied sequentially to improve the sensitivity of ic-CLEIA.

According to the optimized experiment conditions, DAZ and its analogs were detected by ic-ELISA (against Nb-EQ1) and ic-CLEIA (against Nb-biotin). Cross reaction (CR) was defined as follows: CR (%) = (IC_50_ of propiconazole/IC_50_ of analog) × 100.

### 2.5. Samples Analysis

Vegetables of cucumber, lettuce, and cabbage samples were purchased from a local supermarket in Guangzhou, China. They were confirmed by GC-MS to be free of DAZ, then the finely chopped vegetable samples were fortified with DAZ to final concentrations of 3, 5, 10, and 15 ng/g, respectively. A 15 g sample was added to a 50 mL centrifugal tube, followed with the addition of 10 mL of acetonitrile, 4 g of anhydrous magnesium sulfate, 1 g of sodium chloride, 1 g of sodium citrate, 0.5 g of disodium hydrogen citrate, and 1 ceramic homogeneous proton. The sealed tube was shaken violently for 1 min and then centrifuged at 4200 rpm for 5 min. The supernatant was purified by adding 900 mg of anhydrous magnesium sulfate, 150 mg of PSA, and 45 mg of GCB, then it was dried using nitrogen at 40 °C. For the GC-MS test, the sample was redissolved in 1 mL of ethyl acetate and DAZ was determined according to the national food safety standard (GB 23200.113-2018: Determination of 208 pesticides and metabolites residues in foods of plant origin-Gas chromatography-tandem mass spectrometry method). For the ic-CLEIA test, the samples were diluted with PBS at different multiples. Each sample was measured in triplicate.

## 3. Results

### 3.1. Preparation and Characterization of Anti-DAZ Nb

A total of five rounds of immunization were performed on Bactrian camels. The results show that the titer of antiserum significantly increased at third round of immunization (Figure S1). Moreover, the traditional antibody IgG1 and heavy chain antibody IgG2 and IgG3 that was isolated from antiserum by protein A and G columns showed a high affinity to DAZ (inhibition of 39.88% to 52.83%), indicating the successful production of anti-DAZ Nb by Bactrian camel (Figure S2). Based on the affinity to the DAZ consideration, the fourth round of lymphocyte was selected to extract RNA for the subsequent construction of the VHH library.

A complexity of 2.12 × 10^7^ cfu/mL was obtained and used for biopanning against DAZ. Ten clones that were randomly picked from the LB plate were amplified and sequenced; the results indicate that the VHH library has a 100% positive insertion rate and that the sequences were diverse (Figure S3). After four rounds of panning, the titer of the output phages increased from 5 × 10^5^ pfu/mL to 1 × 10^7^ pfu/mL, indicating a significant enrichment of specific phage clones with a binding ability to antigen DAZ-A-BSA. The phage display VHH library titer reached 1.2 × 10^12^ pfu/mL by rescuing with helper phage M13K07. After four rounds of screening, the positive clones were effectively enriched, which guaranteed the subsequent screening of a phage specific to DAZ (Table S2).

**Figure 1:**
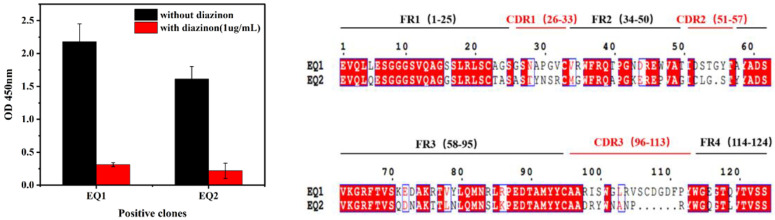
The affinity of two positive phage clones of Nb-EQ1 and Nb-EQ2 and their amino acid sequence alignment.

Notably, the phage-ELISA results showed that 67 out of 96 clones were positive, and they had significant inhibitory rates over 80% for DAZ (1 µg/mL). However, in this study, only two kinds of sequences, named Nb-EQ1 and Nb-EQ2, were found in the positive clones after they were sequenced and aligned, both of which exhibited similar inhibition rates of Nb-EQ1 (85.68%) and Nb-EQ2 (86.52%) with 1 µg/mL DAZ (Figure 1). The simultaneous alignment of their amino acid sequences, Nb-EQ1 and Nb-EQ2, showed a high homology in the framework region but a rich diversity in CDRs. The Nb-EQ1 with the lower IC_50_ value of the ic-ELISA curve was selected for subsequent studies. Nb-EQ1 was expressed in *E. coli* BL21(DE3) and purified via a nickel column; The successful purification of Nb-EQ1 was verified by SDS-PAGE. (Figure S4). The protein yield was about 1 mg/L, and the purity of the target Nb was 95.5%.

### 3.2. Stability and Recognition Mechanism of Nb-EQ1 against DAZ

An ic-ELISA based on the Nb-EQ1 and second antibody of rabbit anti- VHH-HRP was developed to assess the affinity and sensitivity for DAZ. As shown in Figure S5, the absorbance increased as the Nb-EQ1 concentration increased, when the DAZ-A-BSA concentration was 1000 ng/mL. When the concentrations of DAZ-A-BSA and Nb-EQ1 were 1000 ng/mL and 125 ng/mL, respectively, the established ic-ELISA had an IC_50_ of 5.07 ng/mL and an LOD of 1.39 ng/mL. The sensitivity was lower than the ic-ELISA method based on DAZ-mAb [20].

**Figure 2:**
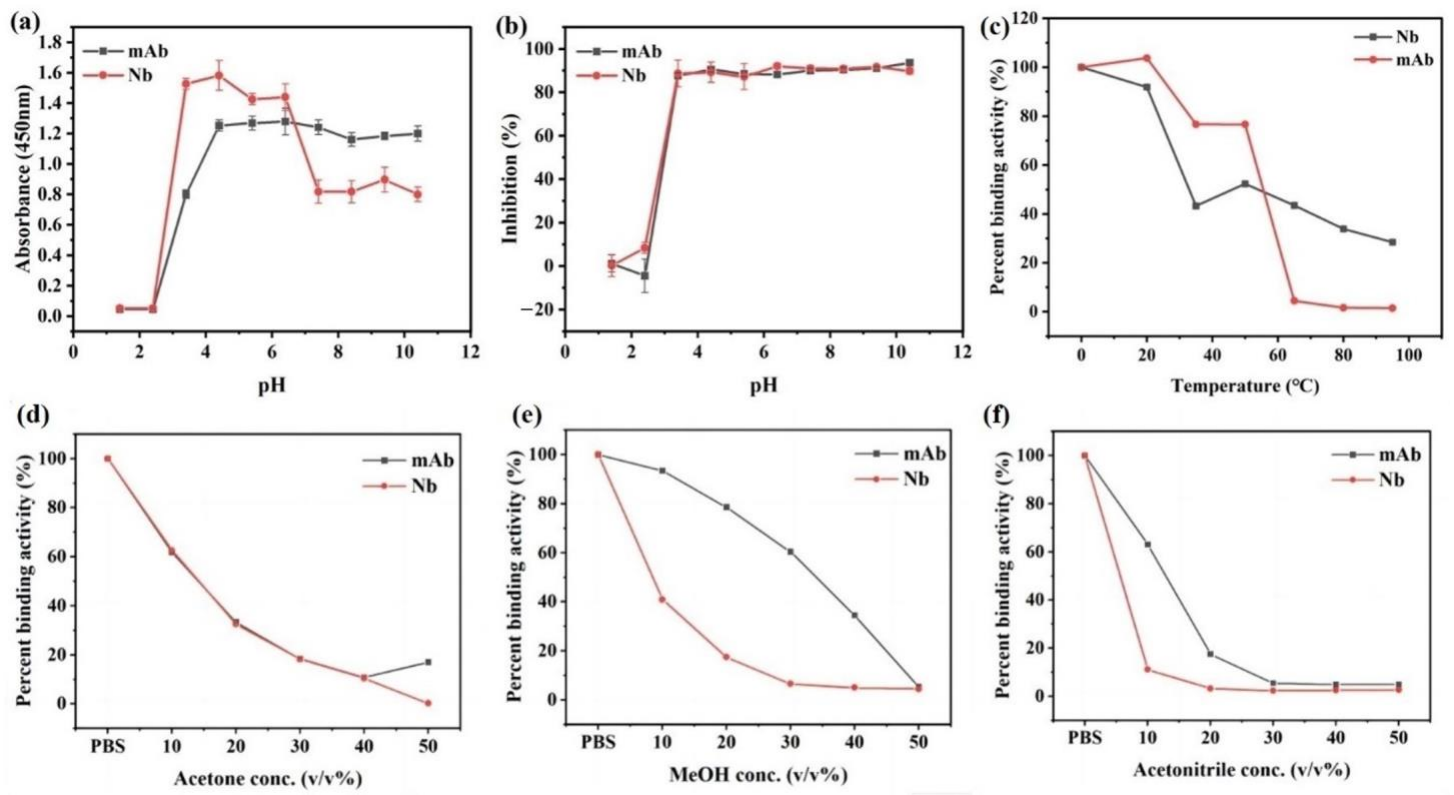
The comparation of stabilities of Nb-EQ1 and DAZ-mAb at (**a**,**b**) different pHs, (**c**) temperatures, and (**d**–**f**) in the presence of an organic solvent.

The stability of Nb-EQ1 at high temperatures, different pHs, and in presence of organic solvents was tested and compared with the DAZ-mAb; the results are shown in Figure 2. The binding activity of Nb-EQ1 against DAZ was superior to DAZ-mAb in the pH range of 3.4–6.4, but there was a significant decrease when the pH exceeded 6. The precent binding activities of Nb-EQ1 against DAZ were lower than that of DAZ-mAb when the temperatures were lower than 50 °C; however, the DAZ-mAb under thermophilic conditions at 65 °C for 5 min was completely inactivated, while Nb-EQ1 remained binding activity of 43.5%. Both the Nb-EQ1 and DAZ-mAb showed a decrease in antigen binding activity at the high concentrate of the organic reagent. Nb-EQ1 possessed a relatively stronger resistance ability than DAZ-mAb at high concentrations of methanol and acetonitrile; it could maintain binding activity of approximately 60% in 30% MeOH and 10% acetonitrile. Generally, compared with mAb, the single-domain structure of Nb made it more stable in harsh conditions [26]. However, the stability of Nb-EQ1 in this study is not significantly superior to DAZ-mAb in an alkali environment and in the presence of organic solvent. This indicates that a high stability is not a universal characteristic of Nb but it rather dependent on special structures, such as the interaction force between crucial amino acid residue and the quantity of disulfide bond [27].

**Figure 3:**
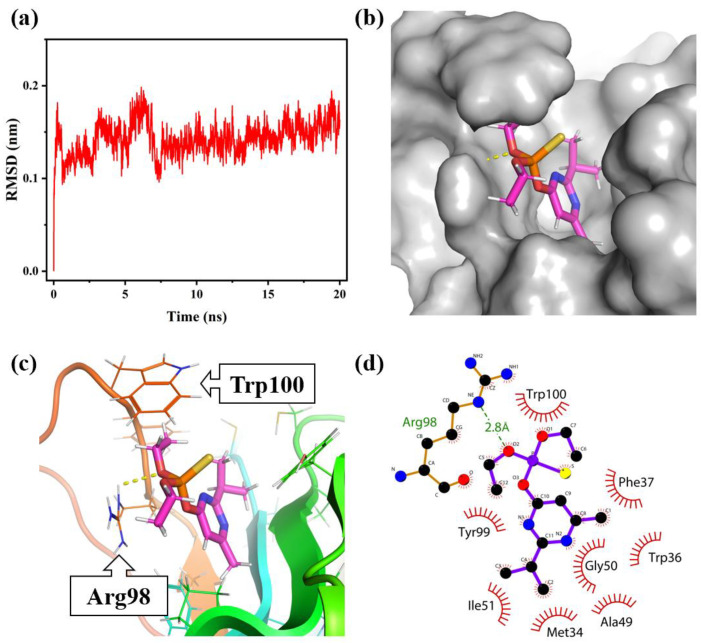
Structure simulation and docking analysis of Nb-EQ1 and DAZ. (**a**) The root-mean-square error; (**b**,**c**) three-dimensional diagram of docking plot; (**d**) two-dimensional diagram of interaction plot.

The recognition mechanism between Nb-EQ1 and DAZ was further performed by molecular modeling with Lead IT 2.3.2-Linux-x64 software. The most accurate Nb-EQ1-DAZ docking state was obtained via dynamic simulation at the minimum energy conformation. As shown in Figure 3a, the root-mean-square error (RMSD) of the Nb-EQ1-DAZ complex reached equilibrium after 20 ns kinetic simulation. Figure 3b,c shows the interaction state of the complex after dynamic equilibrium, where the gray surface is the hydrophobic surface of Nb-EQ1, which results in the formation of a hydrophobic pocket cavity. The hydrophobic pocket is mainly formed by residues such as Tyr99, Ile51, Met34, Ala49, Gly50, Trp36, Phe37, and Trp100 (Figure 3d), most of which are hydrophobic residues. The characteristic group (2-isopropyl-4-methyl-6-pyrimidine) of the DAZ molecule enters this hydrophobic pocket cavity to form a hydrophobic interaction with Nb-EQ1. The thiophosphate group of the DAZ molecule faces outward which forms hydrogen bonds with the antibody (the yellow dotted line in Figure 3b), further strengthening the binding force of Nb-EQ1 and DAZ. Moreover, the Trp100 residue at the top of the pocket cavity forms a “lid” to wrap DAZ, enhancing the affinity of Nb-EQ1 to DAZ. The distance of the hydrogen bond formed by the amino group of Arg98 and the oxygen atom of the DAZ’s thiophosphate group is 2.8 Å, which belongs to a class of hydrogen bonding with a strong bonding force. This indicated that Arg98 might be a key amino acid of Nb-EQ1 in the recognition of the DAZ molecule.

### 3.3. Construction of ic-CLEIA Platform Based on Nb-EQ1 and Streptavidin Recognition

**Scheme 1:**
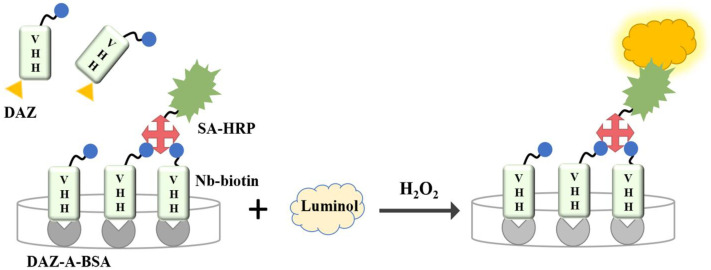
Schematic diagram of ic-CLEIA platform based on Nb-biotin and streptavidin recognition.

The strategy diagram of ic-CLEIA established in this study is shown in Scheme 1. The DAZ and coated antigen DAZ-A-BSA compete for binding with biotinylated Nb-EQ1. Subsequently, the biotinylated Nb-EQ1 conjugates as a coated antigen in the plate, which is bonded with SA-HRP. The ic-CLEIA signal was amplified by biotinylated and streptavidin recognition. Thus, the low level of DAZ in the sample ultimately leads to a high amount of HRP, which finally results in a high readout of the ECL signal.

**Figure 4:**
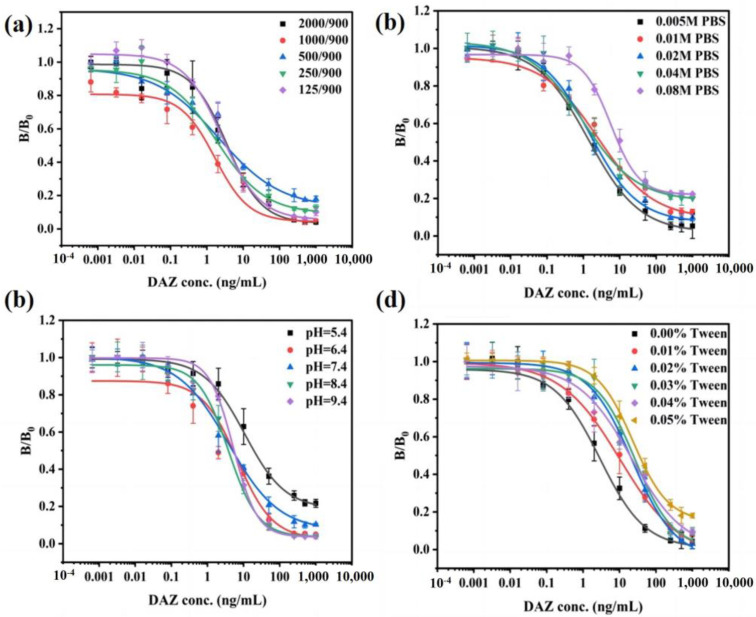
Effect of physicochemical parameters on ic-CLEIA performances. (**a**) Coating antigen/antibody concentration, (**b**) PBS concentration, (**c**) pH value, (**d**) tween-20 concentration.

**Figure 5:**
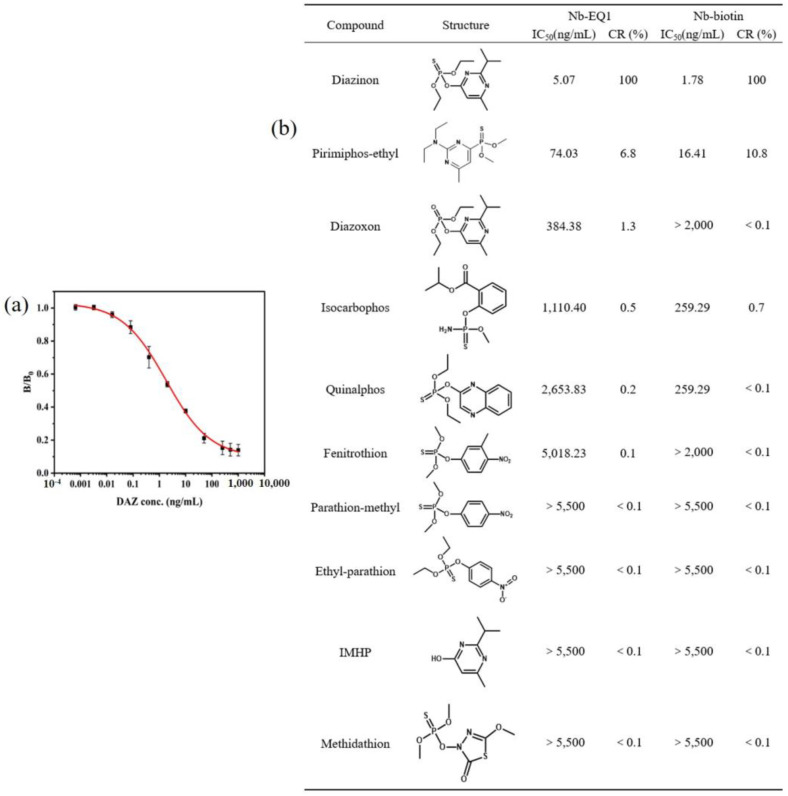
The (**a**) standard curve of DAZ assessed by ic-CLEIA and (**b**) cross-reactivities of the obtained Nb with DAZ and its analogues.

The ic-CLEIA conditions were optimized with respect to the concentration of the coating antigen and biotinylated Nb-EQ1 and the working buffer parameters. On account of the narrow linear range (0.16 ng/mL to 2.10 ng/mL) of ic-ELISA based on mAb, the low IC_50_ value and broad linear range were given priority to evaluate the ic-CLEIA. The results are summarized in Figure 4, where it is shown that a pH of 7.4 and 0.01 M of PBS containing free tween-20 was selected as the working buffer for the development of ic-CLEIA. Under optimal conditions, the standard curve was established by plotting the values of B/B_0_ versus the concentration of DAZ (Figure 5a); the IC_50_ value of the assay was 1.78 ng/mL with an LOD of 0.03 ng/mL. Notably, the linear range was calculated as 0.12–25.96 ng/mL, which possessed approximately a one order of magnitude broader linear range than that in ic-ELISA based on mAb [20] and ic-ELISA based on Nb-EQ1 (Figure S5).

The specificities of the produced Nb-EQ1 (tested by ic-ELISA) and the Nb-biotin (tested by the developed ic-CLEIA) were evaluated using a CR test. As shown in Figure 5b, the Nb-EQ1 showed slight CRs toward ethyl-pirimiphos (6.8%), diazoxon (1.3%), isocarbophos (0.5%), quinalphos (0.2%), and fenitrothion (0.1%), but exhibited unmeasurable CR (CR < 0.1%) with other analogs. Moreover, the Nb-biotin had slight CRs toward ethyl-pirimiphos (10.8%) and isocarbophos (0.7%), respectively, and the CRs toward other DAZ analogues were lower than 0.1%. These results indicated that the produced Nb-EQ1 had a high specificity for DAZ, and the developed ic-CLEIA platform based on Nb-biotin and streptavidin recognition presented a good specificity to DAZ determination.

### 3.4. Samples Analysis

**Figure 6:**
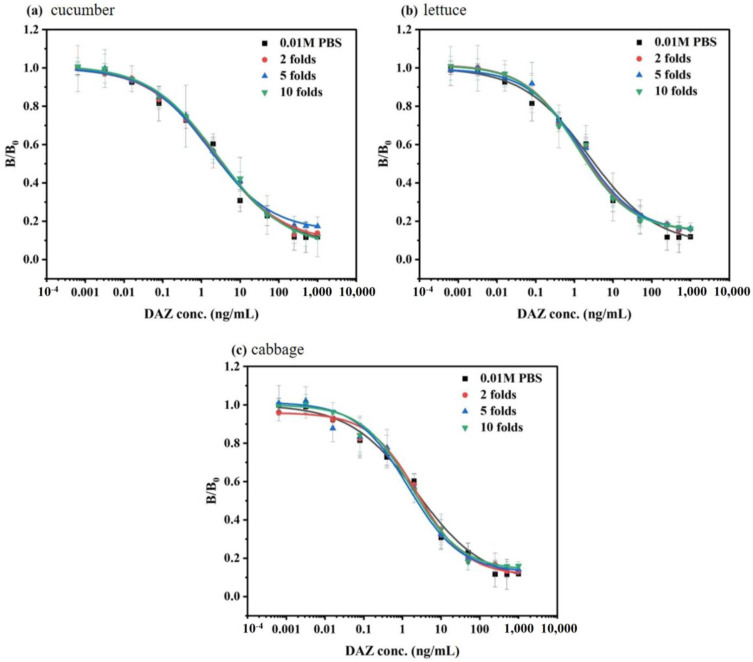
Matrix effect of vegetable samples on the ic-CLEIA performance (*n* = 3); (**a**) cucumber, (**b**) lettuce, (**c**) cabbage.

Matrix and organic reagent residue interference were the common challenges in immunoassays, and it is most commonly eliminated by the dilution of the extract with a working buffer [20,28]. Figure 6 shows the standard curves of cucumber, lettuce, and cabbage; the samples were tested in serially diluted extracts (2-fold, 5-fold, and 10-fold) and 0.01 M PBS (pH 7.4). The standard curve at a 2-fold dilution was similar to the standard curve prepared by the optimal buffer, indicating the lesser effect of the matrix interference in the determination of vegetable samples after QuEChERS preparation. The spiked recoveries of cucumber, lettuce, and cabbage samples are shown in Table 1; the lowest spiked concentration of DAZ in this study was less than or equal to the lowest MRL of DAZ in agricultural products in the European Union (10 μg/kg), the USA (50 μg/kg), and China (10 μg/kg) [20,22]. The average recoveries ranged from 85.7% to 110.9% with variable coefficients of 4.2–19.2%. These results meet the standard of the International Union of Pure and Applied Chemistry (IUPAC, 70–120% with RSD ≤ 20%). In addition, good correlations (R^2^ > 0.97) were obtained between ic-CLEIA and GC-MS (Figure S6), suggesting the good accuracy of the ic-CLEIA based on Nb-EQ1 for the quantitative detection of DAZ in vegetable samples.

## 4. Conclusions

Herein, an ic-CLEIA platform based on the amplification of biotinylated Nb and streptavidin was constructed with a high sensitivity and specificity. Nb-EQ1 with a high sensitivity and specificity against DAZ was first prepared from the phage display library; it exhibited a relatively high thermostability and acid and MeOH stability compared with the corresponding mAb. Nb-Biotin was prepared by conjugating Nb-EQ1 with Sulfo-NHS biotin; the constructed immunosensor showed an LOD of 0.03 ng/mL and a linear range of 0.12–25.96 ng/mL, which had a higher sensitivity and broader linear range than that of ic-ELISA based on mAb and Nb-EQ1. In the determination of cucumber, lettuce, and cabbage, the matrix effects were eliminated by the 2-folds dilution of the extraction solution after QuEChERS purification. The recoveries were 85.7–113.9%, and the CVs were 4.2–19.2%, which was consistent with the results by GC-MS.

## Figures and Tables

**Table 1:** Recoveries of DAZ from spiked vegetable samples by ic-CLEIA.

Sample	Spiked Levels (ng/g)	ic-CLEIA		
		Measured (ng/g)	Recovery (%)	CR (%)
Cucumber	3	3.3 ± 0.3	110.9	9.5
	5	5.4 ± 0.3	108.1	6.4
	10	10.7 ± 1.4	107.1	13.4
	15	14.8 ± 1.3	98.9	8.6
Lettuce	3	3.2 ± 0.6	105.4	19.2
	5	5.4 ± 0.6	107.3	10.7
	10	8.6 ± 0.4	85.7	4.2
	15	15.9 ± 1.2	105.8	7.5
Cabbage	3	3.3 ± 0.4	109.7	13.4
	5	5.4 ± 0.6	107.6	10.5
	10	10.2 ± 1.0	102.1	9.6
	15	15.9 ± 1.1	105.9	6.9

## Data Availability

The data are available on request.

## Supplementary Materials

The following supporting information can be downloaded at: https://www.mdpi.com/article/10.3390/bios13060577/s1. Table S1. The primers for VHH gene amplification. Table S2. Results of affinity panning. Figure S1. Characterization of the Bactrian camel antiserum against free DAZ, (a) the titer curve, and (b) inhibition rate curve; the coating concentration of 1 µg/mL and DAZ content of 1 µg/mL. Figure S2. The characteristic of IgG1, IgG2, and IgG3 isolated the Bactrian camel antiserum ((a) electrophoretogram, (b) the affinity of IgG1, IgG2, and IgG3 against DAZ). Figure S3. Amplified and sequenced results of ten clones that were randomly selected from LB plate. Figure S4. The result of Nb-EQ1 expression and purification. (a) Expressed in *E. coli* BL21(DE3) and plate in LB culture, (b) SDS-PAGE of purified Nb-EQ1. Figure S5. The antigen-binding activity of Nb-EQ1 against DAZ and ic-ELISA standard curve based on Nb-EQ1. Figure S6. Correlation of analysis of samples spiked with parathion between ic-CLEIA based biotinylated Nb-EQ1 and GC-MS.
