# Supplementary material for: A Chemiluminescence Enzyme Immunoassay Based on Biotinylated Nanobody and Streptavidin Amplification for Diazinon Sensitive Quantification
Source: Biosensors (Basel). 2023 May 25;13(6):577. doi: 10.3390/bios13060577 (PMC10296574; doi:10.3390/bios13060577)
Supplement: Supplementary file 1 [file biosensors-13-00577-s001.zip › biosensors-2304990-supplementary.pdf]

Supplementary

A Chemiluminescence Enzyme Immunoassay Based on Biotinylated Nanobody and Streptavidin Amplification for Diazinon Sensitive Quantification

Pengyan Guo [1], Kaiyin Huang [1,2], Zijian Chen [1], Zhenlin Xu [1], Aifen Ou [3], Qingchun Yin [4], Hong Wang [1], Xing Shen [1,*] and Kai Zhou [1,2,*]

[1] Guangdong Provincial Key Laboratory of Food Quality and Safety, South China Agricultural University, Guangzhou 510642, China

[2] Institute of Jiangxi Oil-Tea Camellia, Jiujiang University, Jiujiang 332000, China

[3] School of Food Science and Health Preserving, Guangzhou City Polytechnic, Guangzhou 510006, China

[4] Key Laboratory of Tropical Fruits and Vegetables Quality and Safety for State Market Regulation, Hainan Institute for Food Control, Haikou 570314, China

* Correspondence: shenxing325@163.com (X.S.); zkjy1990@163.com (K.Z.); Tel.: +86-13560432677 (X.S.); +86-13263216525 (K.Z.)

The pComb3xss-VHH plasmid was transferred into *E. coli* BL21(DE)3 competent cells via electrotransformation (GenePulser XcellTM, BIO-RAD), the processes were shown as follows:

(1) 50 μL *E. coli* BL21(DE)3 competent cells were gently mixed with 10μL pComb3xss-VHH plasmid in ice-bath.

(2) the mixture was transferred into 0.2 cm cooled shock cup, and the electrotransformation was set at 2.5 kV.

(3) the cells were resuspended by adding 1 mL SOC medium (preheated at 37°C) and cultured in a shaking bed at 250 rpm at 37°C for 1 h.

(4) the prepared cells were diluted with 10-fold gradient dilution, and then cultured on LB-ampicillin agar plates overnight at 37°C.

(5) the library capacity of the gradient plate was calculated based on plate count.

Table S1. The primers for VHH gene amplification

primer	Sequence
CALL001	5'-GTCCTGGCTGCTCTTCTACAAGG-3'
CALL002	5'-GGTACGTGCTGTTGAACTGTTCC-3'
FR1-SfiI	5'-ACTGGCCCAGGCGGCCGAGGTGCAGCTGSWGSAKTCKG-3'
FR4-SfiI	5'-ACTGGCCGGCCTGGCCTGAGGAGACGGTGACCWGGGTC-3'

Table S2. Results of affinity panning

round	input(pfu)	output(pfu)	Recovery [a]	Enrichment [b]
1	$1.2x10^{12}$	-	$2.4x10^{-5}$	-
2	$1.22x10^{13}$	$3.8x10^{8}$	$3.1x10^{-5}$	1.29
3	$1.43x10^{14}$	$2.88x10^{8}$	$2.01x10^{-6}$	0.06
4	$1.4x10^{14}$	$1.53x10^{10}$	$1x10^{-4}$	50

Note: [a] Recovery =Output titer/Input titer, [b] Enrichment = Output titer of this round/ Output titer of last round

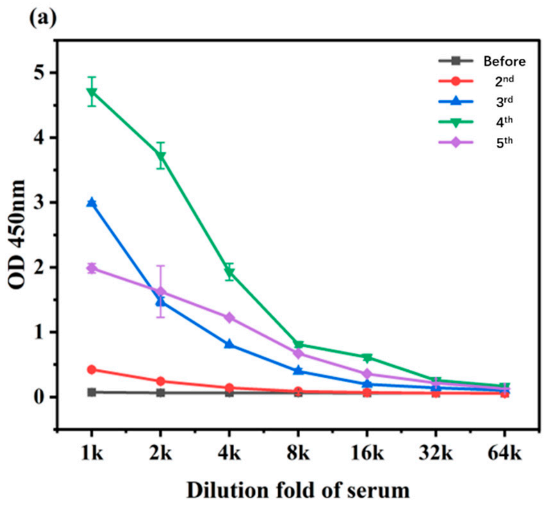

(T)	1K	2K	4K	8K	16K	32K	64K
before	7.69±5.65	-5.51±5.76	9.30±3.17	0.79±7.82	4.92±4.41	0.85±1.18	5.04±2.08
2nd	69.59±1.50	61.03±0.18	48.23±0.45	26.29±4.64	13.77±0.46	4.92±2.09	-1.79±2.53
3rd	80.59±0.63	80.37±2.80	80.05±2.57	73.71 ±2.76	61.64±1.28	45.36±4.80	31.88±3.77
4th	86.63±0.20	89.49±2.16	88.15±0.78	83.62±2.02	80.72±0.95	65.49±1.06	48.79±1.88
5th	78.44±1.99	83.94±4.86	86.37±0.11	83.64±1.62	76.26±0.46	65.44±0.05	46.48±0.32

Figure S1. Characterization of the Bactrian camel antiserum against free DAZ, (**a**) the titer curve, and (T) the inhibition rates (%); the coating concentration of 1 μg/mL and DAZ content of 1 μg/mL

(a)

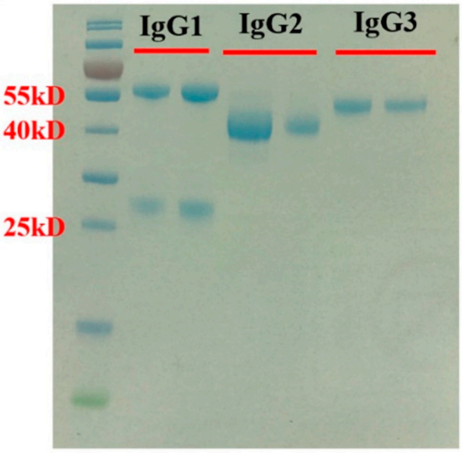

(T)	Titer ($OD_{450\ nm}$)	Inhibition ($OD_{450\ nm}$)	inhibition rate (%)
IgG1	0.92±0.03	0.43±0.02	52.83±0.96
IgG2	1.00±0.02	0.60±0.04	39.88±0.88
IgG3	0.92±0.03	0.47±0.03	48.64±0.89

Figure S2. The characteristic of IgG1, IgG2 and IgG3 isolated the Bactrian camel antiserum ((**a**): electrophoretogram, T: the affinity of IgG1, IgG2 and IgG3 against DAZ).

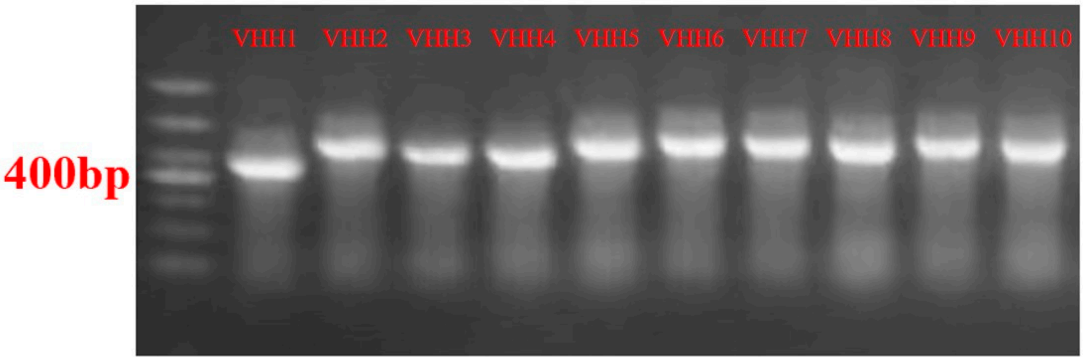

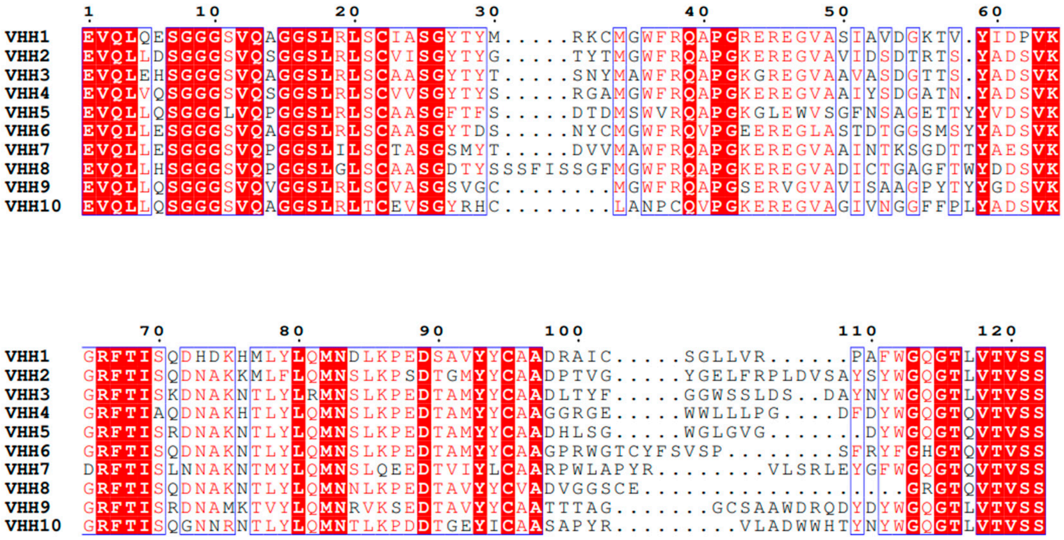

Figure S3. Amplified and sequenced results of ten clones that randomly selected from LB plate.

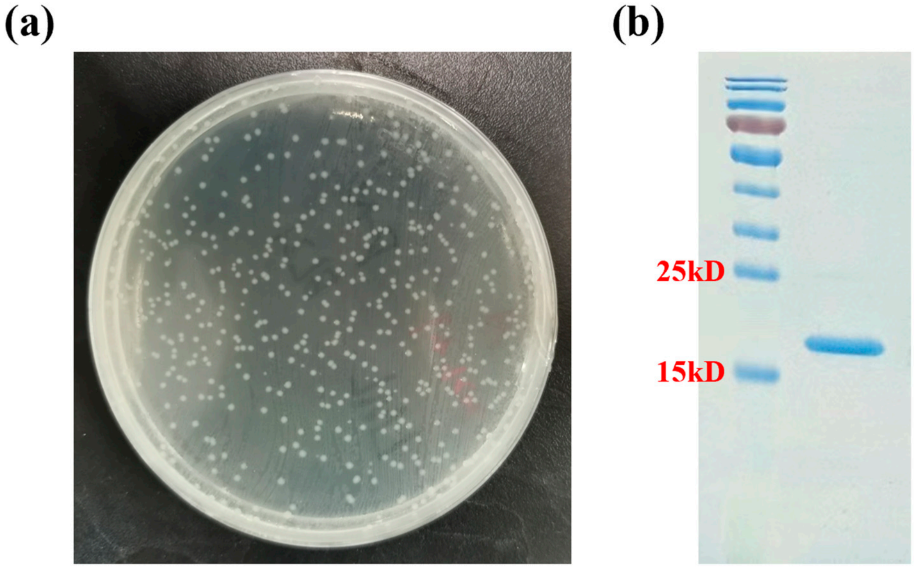

Figure S4. The result of Nb-EQ1 expression and purification. (**a**) the expressed in *E coli* BL21(DE3) and plate in LB culture, (**b**) SDS-PAGE of purified Nb-EQ1.

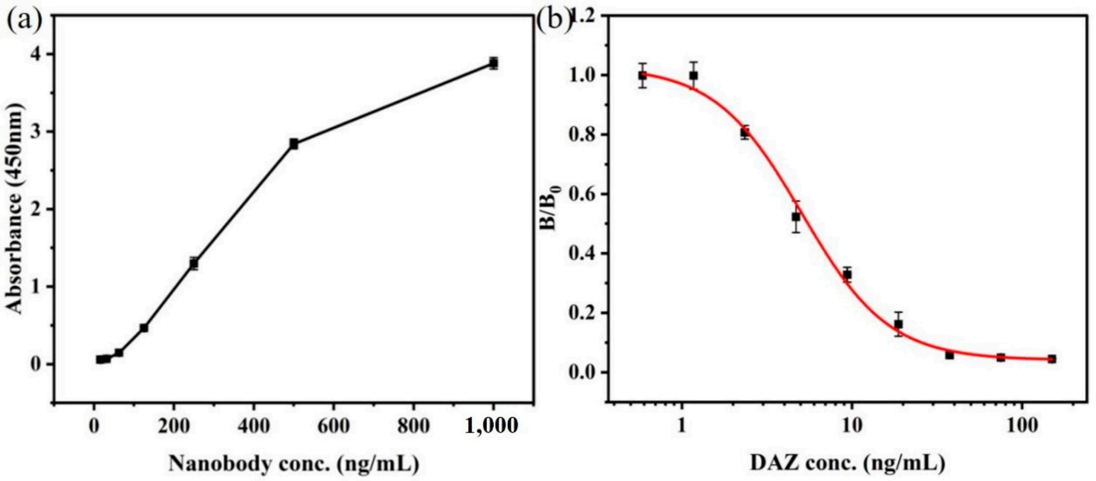

Figure S5. The antigen-binding activity of Nb-EQ1 against DAZ (**a**) and ic-ELISA standard curve based on Nb-EQ1 (**b**).

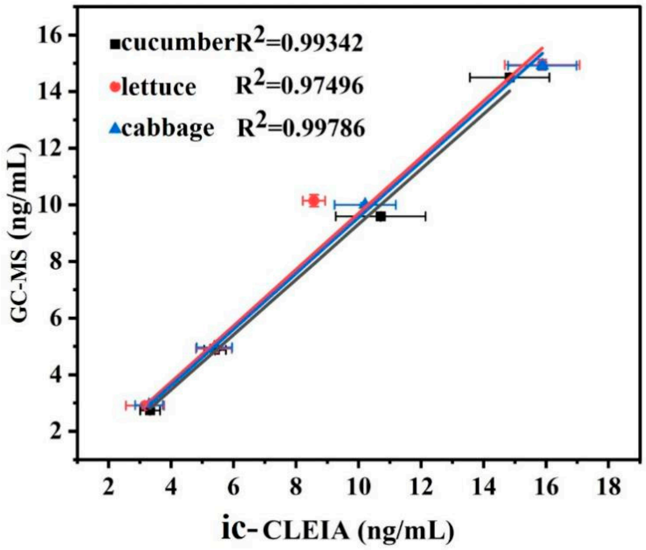

Figure S6. Correlation of analysis of samples spiked with DAZ between ic-CLEIA based biotinylated Nb-EQ1 and GC-MS.
